# Naked cuticle is essential for *Drosophila* wing development beyond Wingless signaling

**DOI:** 10.1002/2211-5463.70149

**Published:** 2025-10-25

**Authors:** Rui Wang, Ping Wang

**Affiliations:** ^1^ School of Engineering Guangzhou College of Technology and Business Guangzhou 510850 China; ^2^ School of Life Science and Technology, MOE Key Laboratory of Developmental Genes and Human Diseases Southeast University Nanjing 210096 China

**Keywords:** development, *Drosophila*, naked cuticle, Wnt

## Abstract

The evolutionarily conserved gene *naked cuticle* (*nkd*) has been gone through studies in both invertebrate and mammalian model systems. Nkd proteins play an essential role in the development of *Drosophila* as a negative‐feedback regulator for Wingless signaling. In this research study, we showcase the multifaceted functions of Nkd in *Drosophila* wing development. The disturbance of *nkd* dosage genetically disrupts multiple biological processes in the larval stage and the morphologies of adult wings. Our results of high‐throughput sequencing indicate that *nkd* may have profound effects on *Drosophila* wing development involving more essential signaling pathways. Nkd may serve as a potential integrator of multiple signaling pathways during wing development, extending its functional repertoire beyond simple Wg inhibition.

## Graphical abstract

Naked cuticle (Nkd), a Wnt signaling inhibitor, assumes extensive roles in *Drosophila* wing development. Overexpressing Nkd causes smaller, crumpled wings, while also perturbing multiple signaling pathways and developmental genes. A specific region (R1S) is critical for Nkd's function as a signaling integrator, offering new insights for studying its homologs in human diseases.

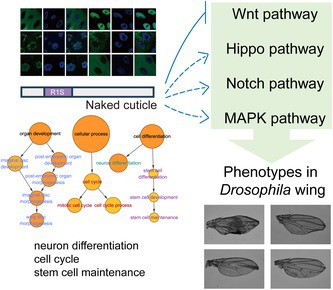

**Abbreviations**
*Ap*
: *Apterous*
*Da*
: *Daughterless*
DEG: differentially expressed gene
Dpp: decapentaplegic
Dsh: dishevelled
FBS: Fetal Bovine Serum
GO: Gene ontology
*Kc*
: *Kc167*
KEGG: Kyoto Encyclopedia of Genes and Genomes
MAPK: mitogen‐activated protein kinase
*nkd*
: *naked cuticle*
NLS: nuclear localization signal
RNA‐seq: RNA sequencing
STF: SuperTopFlash
*wg*
: *wingless*
Wg‐CM: Wg‐conditioned media
WRE: Wnt response element

The development of complex structures like the *Drosophila* wing requires the precise integration of multiple conserved signaling pathways. The interplay between these pathways ensures coordinated cell fate specification, proliferation, and tissue patterning. Therefore, identifying molecules that serve as integrators or nodes for cross talk between these pathways is crucial for understanding the fundamental principles of morphogenesis. Transducers, some of which serve as nodes for cross talk between pathways, are commonly essential for morphogenesis during development and elaborate tissue‐specific gene regulation. Given the crucial role of the Wnt pathway in stem cell development and maintenance [1, 2, 3], it is unsurprising that mutations in Wnt signaling transducers are frequently observed in diseases, including carcinomas. The most well‐characterized example is the APC mutation in colon cancer [4, 5, 6]. *AXIN1*, *AXIN2*, and *β‐catenin* mutations have also been reported in a variety of carcinomas as well [7, 8, 9, 10]. Several groups of molecules physiologically attenuate Wnt signaling through distinct mechanisms. Notably, negative‐feedback regulators, such as Notum, Nemo, and Naked cuticle (Nkd), ensure fine balancing of Wnt signaling [11, 12, 13].

Endogenous *nkd* expression depends on *wingless* (*wg*) [12] and the recruitment of Wg‐dependent TCF to the *nkd* control region has been verified [14], providing evidence that *nkd* is a direct target gene of Wnt signaling. Later studies demonstrated that the inhibitory function of Nkd depends on specific motifs conferring membrane localization, Dishevelled (Dsh) binding, and two potential nuclear localization signals (NLSs) [15, 16, 17, 18, 19]. Aggregation of a conserved C‐terminal histidine cluster has been proven crucial for promoting Axin destabilization [20]. Nkd physically interacts with Dsh, which plays a vital role in both canonical and noncanonical Wnt pathways [21].

Beyond its role in development, the function of Nkd proteins is highly conserved and critically important in human disease. In mammalian species, including humans, there are two *NKD* homologs, *NKD1* and *NKD2* [22], which have emerged as important regulators in cancer biology. Studies have identified and suggested that both *NKD1* [23, 24, 25] and *NKD2* [26, 27, 28] are associated with several human cancers, including colorectal adenocarcinoma and non‐small‐cell lung cancer, by mutations and DNA hypermethylation mediated downregulation. Loss of either *NKD1* or *NKD2* would promote tumorigenesis and/or result in a poor prognosis of cancer. These studies lead to a potential diagnostic application that the methylation or expression of *NKD1/2* might be used as a prognostic marker in cancer. These studies underscore the evolutionary significance of Nkd family proteins and highlight the necessity of understanding their precise molecular functions, which can be elegantly modeled in *Drosophila*. It is important to understand the pleiotropic role of *nkd* as the connection between *nkd* homologs and human diseases.

In this study, we demonstrate that *nkd* impacts multiple biological processes and mediates cross talk between Wnt/Wg signaling and other development‐related pathways during *Drosophila* wing development, largely dependent on Nkd‐Dsh interaction.

## Materials and methods

### *Drosophila* genetics

P[*UAS‐GFP*], P[*UAS‐mCD8::GFP*] [29], P[*UAS‐GFP::lacZ.nls*] [30], P[*UAS‐Nkd* ^*GFPC*^], P[*UAS‐Nkd* ^*ΔR1S/GFPC*^], P[*UAS‐Nkd* ^*Δ30aa/GFPC*^] [17], P[*UAS‐Nkd* ^*ΔD6/GFPC*^] [19], and P[*dpp‐LacZ*] [31] have been described previously and were obtained from the Bloomington Stock Center. All *UAS‐Nkd* transgenic lines were systematically outcrossed into a common, uniform genetic background for at least five generations. Following outcrossing, stable stocks for each transgene were established through re‐balancing with the appropriate chromosome balancers.

All lines were cultured with standard medium at 25 °C. Experiments with *Daughterless‐Gal4*, *Engrailed‐Gal4*, *Apterous‐Gal4*, and *MS1096‐Gal4* were carried out at 25 °C. Larvae were dissected 120 h after egg laying. Adult flies were frozen overnight and photographed with a Nikon ShuttlePix P‐400Rv microscope.

### Antibodies

Immunostaining of imaginal disks was performed as described [32] using the following antibodies: anti‐β‐galactosidase antibodies (1 : 500, Cappel, MP Biomedicals, Santa Ana, CA, USA) and anti‐Wingless (1 : 200, Developmental Studies Hybridoma Bank at the University of Iowa, USA).

### Vectors

*Nkd* alleles expression vectors were generated by subcloning *Nkd* fragments with *GFP* tags at the N terminus into a pAc5.1 expression vector (Invitrogen, Carlsbad, CA, USA). The primer sequences used for plasmid construction are listed below:

*Nkd‐full* forward: 5′‐GGTACCATGGCGGGTAACATTGTCAAATG‐3′, *Nkd‐full* reverse: 5′‐CTCGAGGACATCCTGCTGCTCCTTG‐3′, *Nkd‐del‐R1S* forward: 5′‐CTGGAGGAATTCTCCCGGGCGGAGCAGTGC‐3′, *Nkd‐del‐R1S* reverse: 5′‐CTCCGCCCGGGAGAATTCCTCCAGTCGGAT‐3′, *Nkd‐del‐D6* forward: 5′‐AACCATCAAATGGCCTGCCCGAATCGCCAT‐3′, *Nkd‐del‐D6* reverse: 5′‐ATTCGGGCAGGCCATTTGATGGTTCGGGTG‐3′, *Nkd‐del‐30aa* forward: 5′‐CGAAAATCGGCCGGAAAACCCCAAGCCAAT‐3′, *Nkd‐del‐30aa* reverse: 5′‐TTGGGGTTTTCCGGCCGATTTTCGCTGCTG‐3′.

### *Drosophila* cell culture

*Kc167* (*Kc*) cells were routinely cultured in Schneider's *Drosophila* media (Invitrogen) containing 5% Fetal Bovine Serum (FBS) at 25 °C. Transient transfections were carried out with X‐tremeGENE HP DNA Transfection Reagent (Roche Applied Science, Penzberg, Germany) according to the manufacturer's instructions.

For the Wnt response element (WRE) reporter assay, a mixture of DNA containing 100 ng *SuperTopFlash* (*STF*) vectors, 200 ng of ectopic *Nkd* alleles expression vectors, and 1 ng *pAc‐lacZ* (Invitrogen) was co‐transfected into 10^6^ cells. Transfection efficiency was normalized using the pAc‐lacZ β‐galactosidase activities. Luciferase and β‐galactosidase activities were assayed using the Tropix Luc‐Screen and Galacto‐Star kits (Applied Biosystems, Carlsbad, CA, USA) and quantitated with a Chameleon plate luminometer (Hidex Personal Life Science, Turku, Finland).

*pTub‐wg S2* cells were kindly provided by Dr Roel Nusse from Stanford University. *pTub‐wg S2* cells were cultured in Schneider's *Drosophila* media (Invitrogen) containing 10% FBS at 25 °C. Wg‐conditioned media (Wg‐CM) was concentrated and stored as described [14]. *Kc* cells were treated with Wg‐CM for 12 h prior to harvesting.

### RNA sequencing analysis

Each RNA sample was prepared in biological duplicate from ~ 70 wing imaginal disks of late third larvae expressing GFP or Nkd recombinant proteins under the control of *MS1096‐Gal4*, which has been shown to express GAL4 in the central pouch region of wing disks [33, 34, 35]. Total RNA was extracted using TRIzol (Invitrogen). RNA‐seq libraries were generated using NEBNext UltraTM RNA Library Prep Kit for Illumina (NEB, Ipswich, MA, USA) following manufacturer's recommendations. RNA‐seq was performed by using Illumina NovaSeq 6000 platform and 150‐bp paired‐end reads were generated. Paired‐end clean reads were aligned to the fly reference genome (BDGP6) with hisat2 (version 2.2.1) [36]. The aligned reads were used to quantify mRNA expression by using featureCounts. The deseq2 package in r [37] was used to compute normalized fold change and adjusted *P* value for each gene, comparing Nkd recombinant protein‐expressing wing disks to the GFP‐expressing controls. Those genes with |fold change| > 1.5 and adjusted *P* value < 0.05 were considered to be differentially expressed. For bioinformatic analyses, the volcano plot showing differentially expressed genes (DEGs) was generated with ggplot2 package in r. Gene Ontology (GO) enrichment analysis of those DEGs was performed using david v6.8 [38, 39] and cytoscape v3.6.1 [40] with a bingo plugin (v3.0.3) [41, 42]. Kyoto Encyclopedia of Genes and Genomes (KEGG) analyses were carried out with online software kobas 3.0 [43]. The RNA‐seq reads used for this study have been submitted to the National Center for Biotechnology Information (NCBI) under Database ID: GSE167145.

## Results

### Overexpression of mutant Nkd proteins suppresses Wg pathway in Kc167 cells

**Fig. 1:**
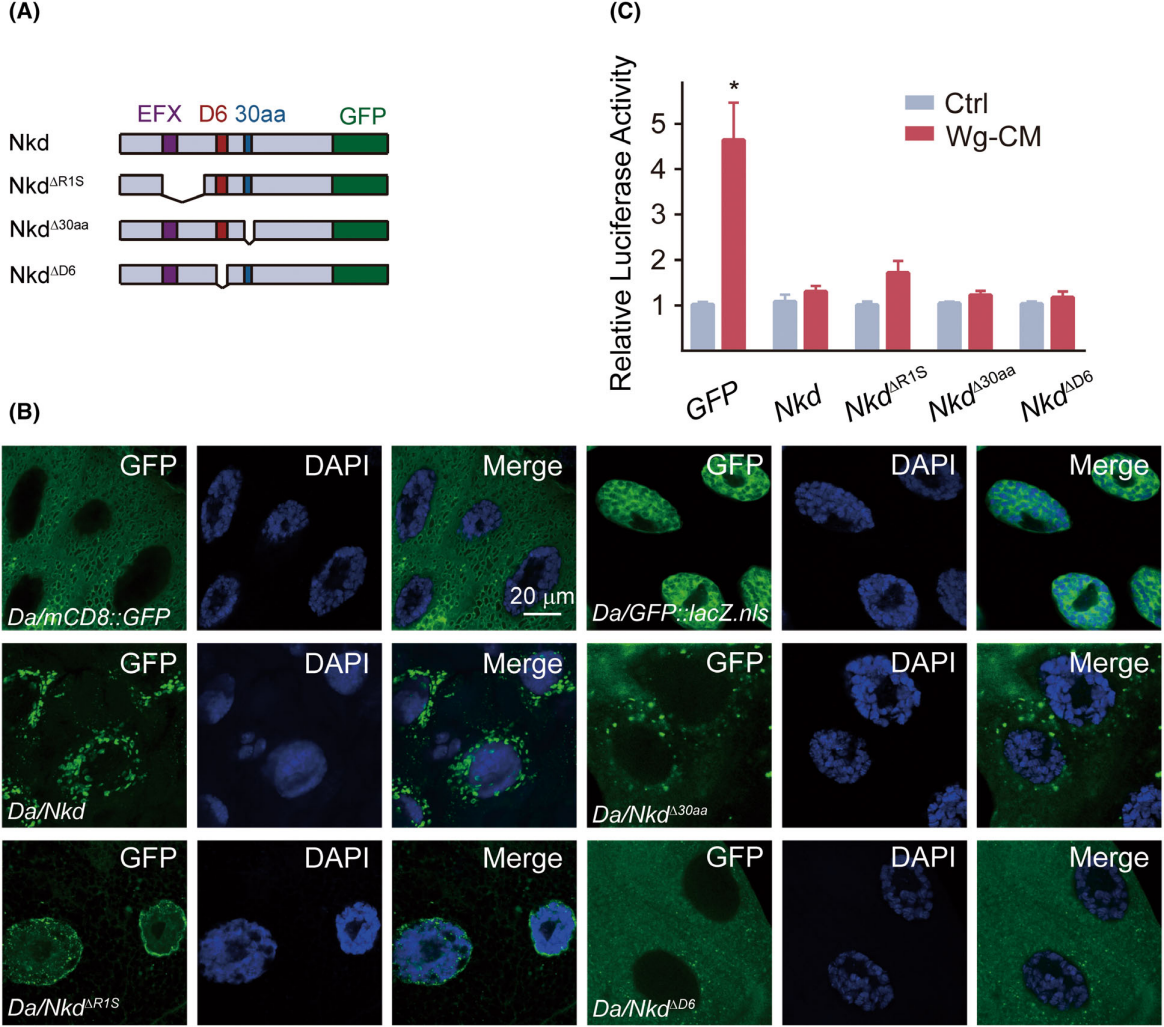
Nuclear localization of Nkd in the salivary gland requires the D6 motif and a 30 amino acid fragment. (A) A diagram showing the domain structures of ectopic Nkd mutants to be overexpressed in flies. (B) Representative confocal images of the late third instar salivary gland cells with genotypes as indicated. Nuclei are stained with DAPI (blue). Scale bar is 20 μm. (C) SuperTopFlash reporter assay measuring Wg pathway activity. Kc cells treated with Wg‐CM show suppressed luciferase activity across all Nkd mutants. Experiments were performed in triplicate and repeated at least three times. The data are expressed as mean ± SEM. * indicates *P* < 0.05 compared with the control group, as analyzed by ANOVA, followed by Tukey's multiple comparison *post hoc* test.

In order to gain further insight into the Nkd function and subcellular localization, we sought to use several published *UAS* alleles of *nkd*, including a full‐length wild‐type *Nkd*, P[*UAS‐Nkd*] [17], and three mutant alleles of *Nkd*. *Nkd* ^ΔR1S^ lacks the Dsh binding region [16]. *Nkd* ^Δ30aa^ and *Nkd* ^ΔD6^ have potential nuclear localization signals (NLSs) deleted [17, 19] (Fig. 1A). Using the *Drosophila* salivary gland system, we examined the subcellular localization of Nkd mutants. These mutants were ubiquitously overexpressed using the *Daughterless‐Gal4* (*Da‐Gal4*) driver. Membrane‐localized and nuclear‐localized GFP served as controls (Fig. 1B). Wild‐type Nkd accumulated around the nuclear periphery with partial intranuclear puncta. Both *Nkd* ^Δ30aa^ and *Nkd* ^ΔD6^ localized cytoplasmically, while *Nkd* ^ΔR1S^ predominantly accumulated in the nucleus. This indicates that Nkd nuclear localization requires both putative NLSs [19]. Notably, the *Nkd* ^ΔR1S^ mutant exhibits preferential nuclear accumulation. Due to limitations of larval salivary glands (polytene, nondiploid cells) for functional insights, we generated Nkd mutant constructs based on *in vivo* fragments and investigated their function in *Drosophila* Kc cells via reporter assays.

The WRE serves as an indirect indicator of cellular Wg signaling intensity. SuperTopFlash (STF) comprises 12 tandem TCF‐binding sites [14]. Upon Wg pathway activation, this reporter plasmid drives luciferase expression, allowing semi‐quantitative measurement of Wg signaling via luciferase activity. Treatment with Wg‐conditioned media (Wg‐CM) activated the Wg pathway in GFP‐overexpressing control cells. Reporter assays revealed that all Nkd mutants suppressed Wg signaling upon Wg‐CM stimulation (Fig. 1C).

However, reporter assays may arise discrepancies with variable expression levels and differential cellular contexts. Adult wing phenotypic analysis *in vivo* may yield more biologically meaningful insights.

### Nkd overexpression modulates wing morphogenesis

**Fig. 2:**
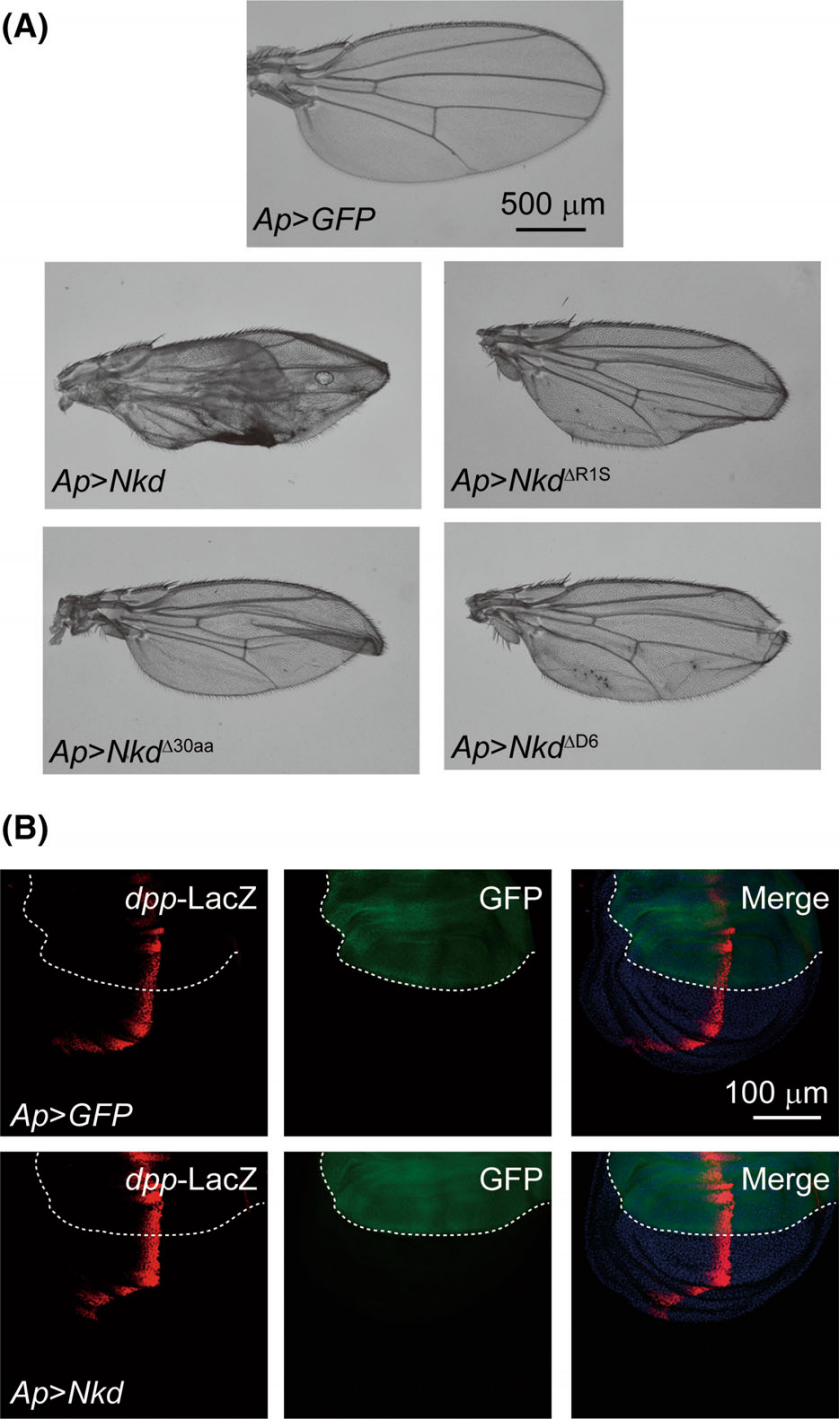
Nkd overexpression modulates wing morphogenesis without altering Dpp expression pattern. (A) Representative images of female adult wings from flies with genotypes as indicated. Scale bar is 500 μm. (B) Representative confocal images of the third instar larval wing disks with antibodies against β‐galactosidase (LacZ, red) with genotypes as indicated. Dorsal/ventral boundaries are marked by the white dotted lines. Nuclei stained with DAPI (blue). Scale bar is 100 μm.

The crumpled wings and necrotic‐like appearance were frequently observed when either *Nkd* or mutant alleles were driven by *Apterous‐Gal4* (*Ap‐Gal4*) in the ventral wing pouch (Fig. 2A). In addition to morphological alterations, the wings of mutant flies exhibited size reduction compared with those of *Ap‐Gal4* > *GFP* controls.

During development, graded morphogens regulate gene expression across fields of cells in a concentration‐dependent manner [44]. Ectopic overexpression or insufficient expression of Decapentaplegic (Dpp) correspondingly alters imaginal disk size [45]. Given the established correlation between Dpp signaling strength and wing size, we investigated whether observed wing size variations result from altered Dpp expression levels. In our experiments, ectopic Nkd expression in the *Drosophila* wing disk induced no significant change in Dpp expression pattern (Fig. 2B). This suggests that Dpp occupies a position upstream of Nkd within the wing developmental regulatory network. These results prompted us to hypothesize that the role of *nkd* function in wing development might be beyond Wingless signaling, consistent with our understanding that *Drosophila* wing development is orchestrated by many signaling pathways.

### Overproduction of Nkd disrupts transcriptional networks in wing disks

**Fig. 3:**
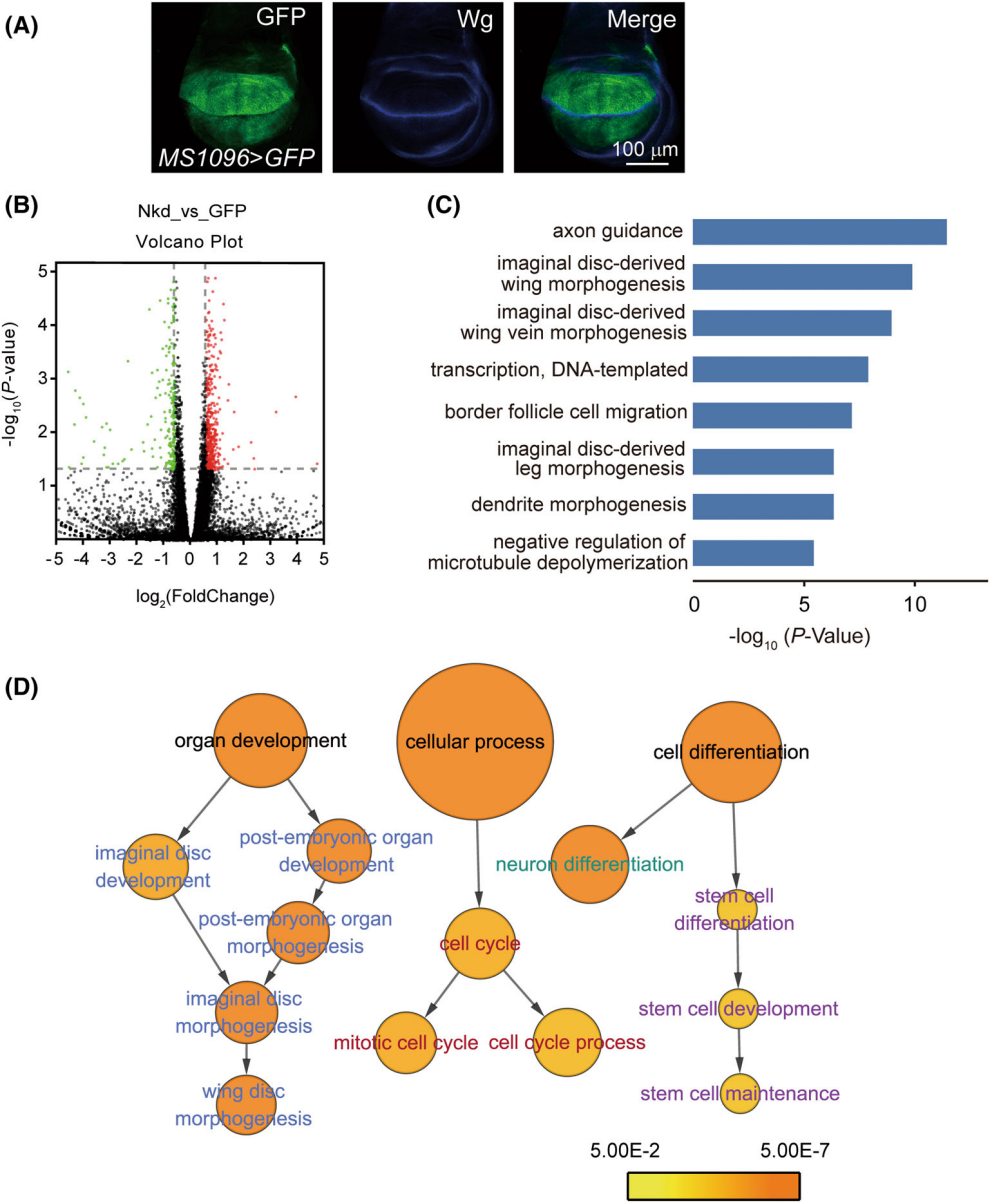
Overproduction of *Nkd* in wing disks affects biological processes in larvae. (A) Representative confocal images of the late third instar wing imaginal disks stained with antibodies against Wingless (Wg, blue) with genotypes indicated. Scale bar is 100 μm. (B) The differentially expressed genes (DEGs) were illustrated in volcano plots (|fold change| > 1.5 and adjusted *P* value < 0.05). Data points in red represent upregulated and green represent downregulated genes. Genes without any significant difference are in black. (C) Functional annotation clustering analysis of differentially expressed genes derived from *Nkd* overexpression was performed using DAVID. (D) Gene Ontology (GO) charts for Biological Processes of DEGs between Nkd group and GFP control, analyzed and visualized as directed acyclic graphs by Cytoscape. GO categories are shown in circles with the area proportional to the ratios of observed gene frequencies over expected ones and the intensities of the orange color gradient correlate with minus Log_10_(*P* values).

To explore the *nkd* function in the wing at a molecular level, we examined the whole transcriptional profiles of wing disks expressing Nkd mutants and GFP controls by performing RNA sequencing (RNA‐seq) analysis. *MS1096‐Gal4* was reported to express GAL4 in the central pouch region of third instar larval wing disks [33, 34, 35] (Fig. 3A), which could induce Nkd expression in a broader manner than *En‐Gal4* and *Ap‐Gal4* do. RNA samples were extracted from dissected wing primordia expressing Nkd proteins driven by *MS1096‐Gal4* (see Materials and methods).

The DEGs between Nkd group and GFP control (Nkd_vs._GFP) include 418 upregulated genes and 254 downregulated genes (|fold change| > 1.5 and adjusted *P* value < 0.05) (Fig. 3B). Overexpression of Nkd significantly impacts the expression of multiple genes involved in wing morphogenesis. Functional annotation clustering analysis revealed significant enrichment of biological processes critical for *Drosophila* wing development, including imaginal disk‐derived wing morphogenesis and wing vein specification. Key transcriptional regulatory pathways, such as DNA‐templated transcription, were concurrently enriched, suggesting coordinated genetic control of wing patterning (Fig. 3C). Gene Ontology analysis performed by Cytoscape detailed these DEGs in some interesting biological processes, such as postembryonic organ development, cell cycle, neuron differentiation, and stem cell maintenance (Fig. 3D). These processes play crucial roles in *Drosophila* wing development, determining wing shape, vein patterning, and transcriptional regulation.

### R1S region is essential for Nkd‐mediated signaling cross talk

**Fig. 4:**
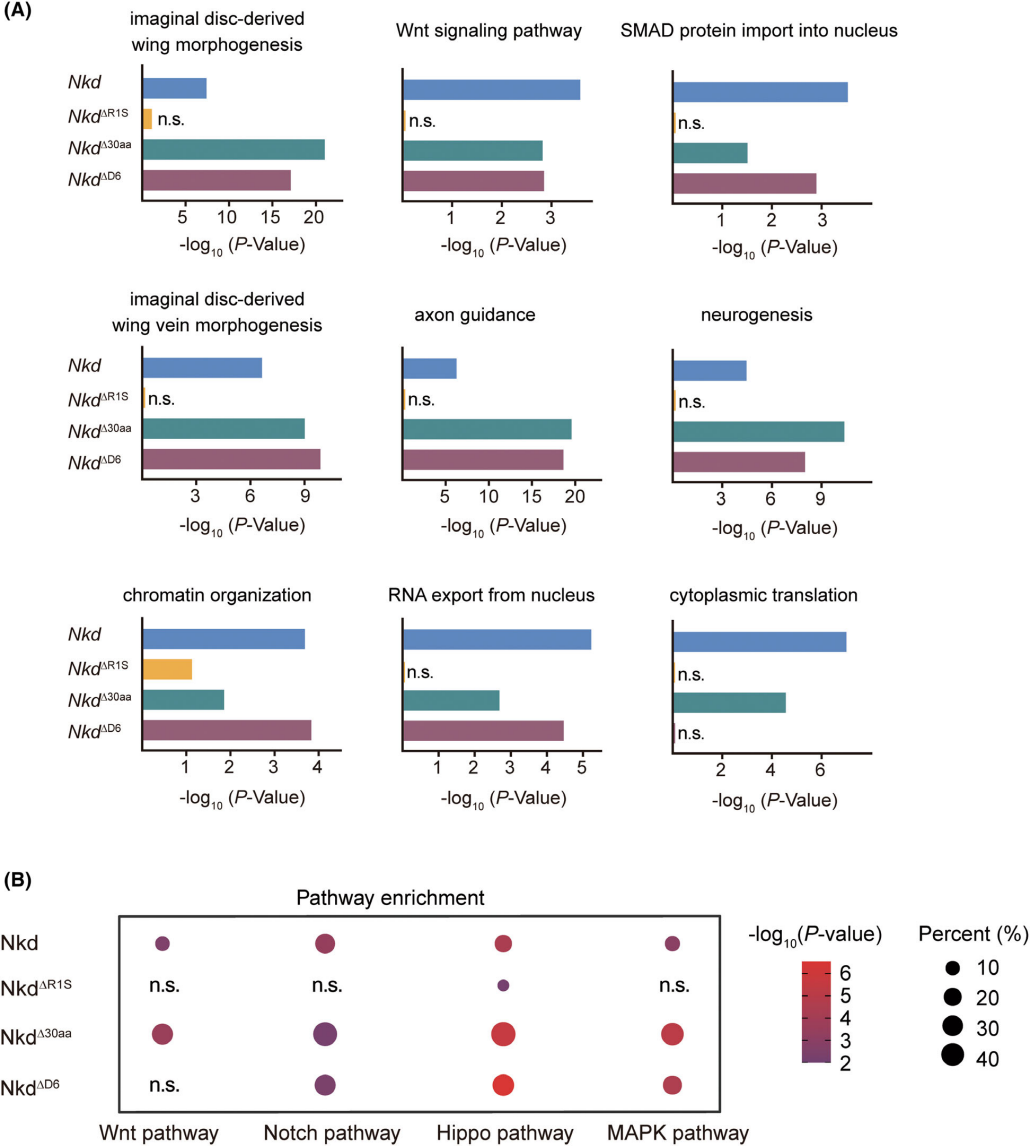
Overproduction of Nkd in wing disks disturbs multiple biological processes and signalings involved in *Drosophila* development. (A) Top biological process terms enriched in DAVID functional Category analysis of DEGs in different groups. n.s., not significant. (B) The bubble chart showing enriched DEGs in KEGG signaling pathway for different groups. The color and size of the bubble represent *P* value and the percentage of differentially expressed genes enriched in the pathway, respectively.

For better understanding of Nkd function in biological processes, we analyzed DEGs between different Nkd mutant groups and GFP control with the same threshold. We processed DEGs in each group with david functional category analysis and found that these genes were enriched in imaginal disk derived wing morphogenesis, neurogenesis as well as other biological processes, including chromatin organization (Fig. 4A), which suggests that Nkd participates in wing development from many directions directly or indirectly.

The expression of some key components in Wnt/Wg signaling and/or other pathways has changed significantly when Nkd is overproduced. KEGG pathway analysis revealed that these differentially expressed genes were highly involved in several critical pathways, including Hippo, Notch, mitogen‐activated protein kinase (MAPK), and Wnt signaling pathways (Fig. 4B and Table 1). While *Nkd* ^ΔR1S^ overexpression altered Hippo signaling, it minimally affected Wnt, Notch, and MAPK pathways or processes like wing morphogenesis compared with wild‐type Nkd (Fig. 4B). This result could be attributed to the limited number of DEGs in the Nkd^ΔR1S^_vs._GFP group (116 DEGs). These data prompt us to the conclusion that the R1S region is crucial for Nkd in development‐related signaling pathways, not merely Wnt signaling. The mediated role of Nkd in cross talk between Wnt/Wg signaling and other development‐related pathways in *Drosophila* largely depends on the R1S region.

**Table 1:** List of Nkd_vs._GFP DEGs significantly enriched in Hippo, Notch, MAPK, and Wnt signaling pathways.

Term	*P* value	Genes
Wnt signaling pathway	0.002976	*Seven in absentia* (*sina*); *Protein kinase, cAMP‐dependent, catalytic subunit 1* (*Pka‐C1*); *Myc*; *arrow* (*arr*); *nkd*; *sarcoplasmic calcium‐binding protein* (*CBP*); *groucho* (*gro*); *dally‐like* (*dlp*); *pangolin* (*pan*); *division abnormally delayed* (*dally*); *Adenomatous polyposis coli tumor suppressor homolog 2* (*Apc2*)
Notch signaling pathway	0.006327	*Notch* (*N*); *Serrate* (*Ser*); *CBP*; *gro*; *presenilin enhancer* (*pen‐2*); *Delta* (*Dl*)
Hippo signaling pathway	0.001121	*Dally*; *disks large 1* (*dlg1*); *Myc*; *echinoid* (*ed*); *bazooka* (*baz*); *lowfat* (*lft*); *crumbs* (*crb*); *Grunge* (*Gug*); *dachs* (*d*); *vein* (*vn*); *fat* (*ft*)
MAPK signaling pathway	0.002976	*Capicua* (*cic*); *puckered* (*puc*); *Raf oncogene* (*Raf*); *embargoed* (*emb*); *sina*; *Epidermal growth factor receptor* (*Egfr*); *sprouty* (*sty*); *Son of sevenless* (*Sos*); *gro*; *vn*; *mitogen‐activated protein kinase kinase kinase* (*Mekk1*)

## Discussion

Wnt signaling is most famous for the ability to shape growing tissues while inducing proliferation in development [1, 3]. Wnt signaling should be finely balanced as it is crucial for the development and maintenance of stem cells. Nkd proteins play an essential role in Wnt/Wg signaling as a negative‐feedback regulator.

Our research demonstrates that Nkd exerts pleiotropic effects on *Drosophila* wing development, extending beyond its canonical role as a Wnt/Wg signaling inhibitor. Nkd overexpression induced wing size reduction and crumpling, which indicates there are more features for *nkd* or its homologs beyond inhibiting Wg signaling [46, 47, 48, 49].

Interestingly, while ectopic Nkd expression induced a pronounced reduction in wing size, it did not alter the expression pattern of Dpp, a key morphogen governing wing growth. This indicates that the size defect is not mediated through the canonical Dpp signaling gradient. Instead, we propose that the observed growth impairment likely stems from the broad transcriptional perturbations revealed by our RNA‐seq analysis. Specifically, the significant enrichment of DEGs related to cell cycle suggests a mechanism whereby Nkd overexpression may directly impact cell proliferation or survival. Furthermore, the profound disruption of the Hippo signaling pathway presents a particularly compelling alternative mechanism, as Hippo is a well‐established central regulator of organ size that acts in parallel to Dpp. Future studies quantifying cell number, cell size, and apoptosis in the wing disk upon Nkd overexpression will be crucial to precisely distinguish between these possibilities.

RNA‐seq analysis revealed widespread transcriptional perturbations in wing disks overexpressing Nkd, with significant enrichment of genes linked to Hippo, Notch, MAPK, and Wnt pathways. Beyond its developmental implications, our work provides new mechanistic insights into the tumor‐suppressive role of Nkd homologs in human cancers. The observation that Nkd‐mediated cross talk critically depends on the R1S region redefines Nkd proteins from mere Wnt pathway inhibitors to potential signaling integrators. This expanded role has direct implications for diagnostic and therapeutic strategies. For diagnosis, the downregulation or mutation of *NKD1*/*2*, as observed in carcinomas, likely leads to a concurrent dysregulation of a network of oncogenic pathways [23, 24, 25, 26, 27, 28]. This could explain the association of *NKD* loss with poor prognosis and suggests that NKD status could serve as a more comprehensive biomarker for tumor multi‐pathway activity. Therapeutically, our data suggest that restoring the function of NKD may represent a novel strategy to simultaneously dampen multiple oncogenic signals. Future efforts could focus on identifying small molecules that stabilize the NKD‐DVL interaction or otherwise enhance NKD integrative activity, offering a potential multi‐pathway intervention for Wnt‐dysregulated cancers.

It is important to note that the broad transcriptional changes observed upon Nkd overexpression could encompass both direct regulatory targets and secondary consequences of developmental disruption. Future studies employing tissue‐specific knockdown and direct measurement of protein levels of key pathway components will be essential to validate and extend these transcriptomic findings. The DEGs identified here should be considered as a resource that defines the ‘Nkd‐overexpression signature’ and implicates specific pathways. Future work employing tissue‐specific, acute protein degradation systems combined with assays for direct binding will be essential to disentangle the direct targets from the secondary network effects and precisely map the domains responsible for specific interactions. Although variable expression levels of Nkd transgenes preclude quantitative comparison between mutants, the Nkd^ΔR1S^ allele uniquely failed to perturb Notch/MAPK pathways (Fig. 4B). This specific phenotype, which coupled with its nuclear accumulation, highlights the R1S region as a critical determinant for cross talk. The conserved role of R1S in Dsh binding [15, 16] suggests that the integrator function of Nkd may be evolutionarily wired, positioning it as a therapeutic node for Wnt‐dysregulated diseases.

Our data propose that Nkd homologs may serve as signaling nodes integrating multiple pathways. The multifaceted role of Nkd in *Drosophila* wing development highlights its capacity to integrate Wnt signaling with other pathways. These findings expand the functional repertoire of Nkd beyond canonical Wnt inhibition and provide a framework for investigating Nkd homologs in developmental and disease contexts.

## Conflict of interest

The authors declare no conflict of interest.

## Author contributions

RW conceived and designed the project. RW and PW conducted experiments and analyzed results. RW wrote the paper. Both authors reviewed and approved the manuscript.

## Data Availability

The data that support the findings of this study are available from the corresponding author (wangrui69@gzgs.edu.cn) upon reasonable request. RNA‐seq data that support the findings of this study are openly available in NCBI's Gene Expression Omnibus and are accessible through https://www.ncbi.nlm.nih.gov/geo/query/acc.cgi?acc=GSE167145, GEO Series accession number (GSE167145).
